# Supplementary material for: Plasmodium vivax infection compromises reticulocyte stability
Source: Nat Commun. 2021 Mar 12;12:1629. doi: 10.1038/s41467-021-21886-x (PMC7955053; doi:10.1038/s41467-021-21886-x)
Supplement: Supplementary file 1 — Supplementary information [file 41467_2021_21886_MOESM1_ESM.pdf]

Supplementary Information

Plasmodium vivax infection compromises reticulocyte stability

Clark *et al.*

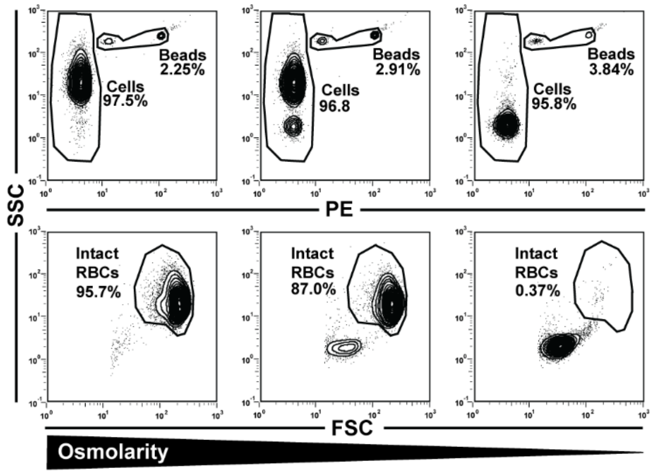

Supplementary Figure 1. Flow cytometry assessment of RBC osmotic lysis.

Representative flow plots distinguishing beads and RBCs (SSC x PE plots) and surviving RBCs and RBC ghosts (SSC x FSC plots) of RBCs recovered from increasingly hypotonic conditions. Data representative of 15 independent experiments.

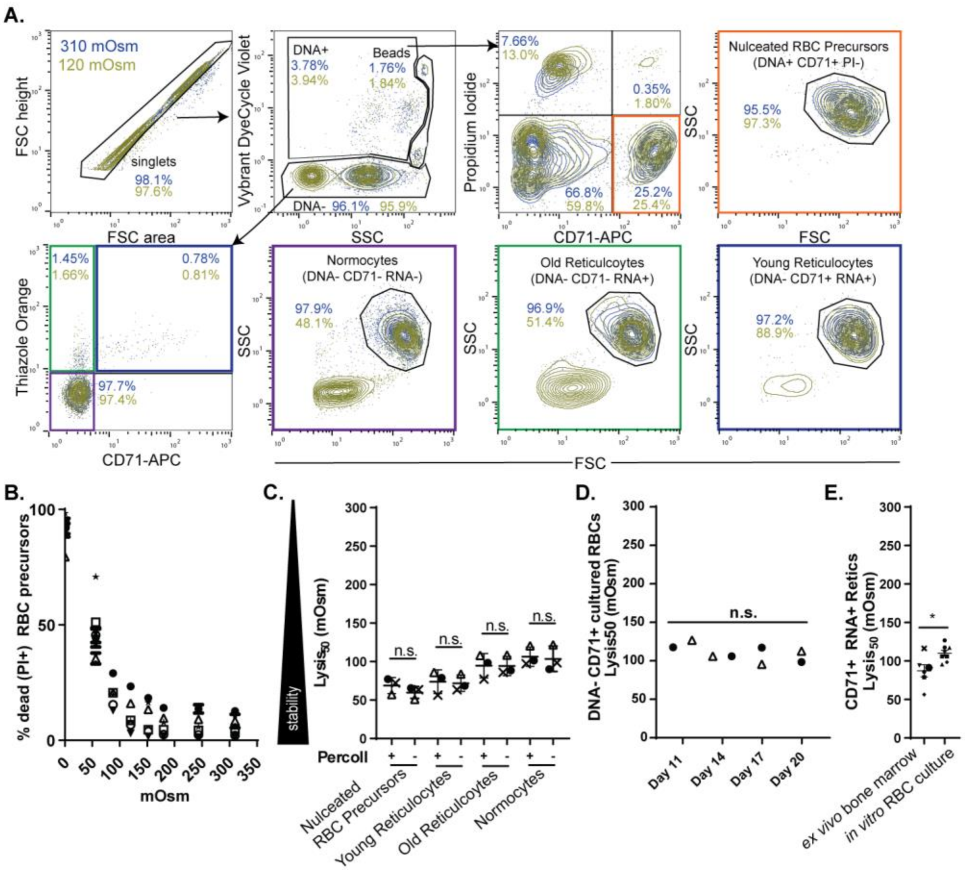

Supplementary Figure 2. Measuring the osmotic stability of reticulocytes and RBC progenitors by flow cytometry. (a) Flow cytometry gating strategy for measuring hemolysis of nucleated RBC precursors (DNA+ CD71+) young reticulocytes (DNA- RNA+ CD71+) old reticulocytes (DNA- RNA+ CD71-) and normocytes (DNA- RNA- CD71-) subpopulations present in bone marrow aspirates. Gating strategy used to assess the osmotic stability of RBC precursors reticulocytes and normocytes from ex vivo bone marrow aspirates and in vitro RBC differentiations as reported in Figure 2. (b) Frequency of dead RBC precursors (DNA+ CD71+) as osmolarity decreases in bone marrow aspirates (n=6). Unique symbol indicate biological replicates. (c) Normocyte (DNA- RNA- CD71-), reticulocyte (DNA- RNA+ CD71- and DNA- RNA+ CD71+), and erythroid precursor (DNA+ CD71+) from bone marrow aspirates lysis$_{50}$ values following

centrifugation in the presence and absence of Percoll (n=3). Unique symbols indicate biological replicates. Horizontal lines and error bars represent mean ±SEM. n.s., no significant difference between the $lysis_{50}$ values of cells centrifuged in the absence and presence of Percoll (nucleated RBC precursors p=0.6, young reticulocytes p=0.1, old reticulocytes p=0.7, normocytes p=0.1) using paired two-tailed student's *t*-test. (d) CD71+ reticulocyte differentiated in vitro from CD34+ stem cells $Lysis_{50}$ values (n=2). Unique symbols indicate the $lysis_{50}$ values at days 11, 14, 17 and 20 of two independent differentiations. n.s., no significant difference in CD71+ reticulocyte $lysis_{50}$ values during differentiation (p=0.5) using Friedman test. (e) Ex vivo bone marrow aspirate CD71+ reticulocytes and in vitro differentiated CD71+ reticulocyte $lysis_{50}$ values. Unique symbols for *ex vivo* bone marrow aspirate CD71+ reticulocytes represent biological replicates (n=6). Unique symbols for in vitro differentiated CD71+ reticulocytes correspond to the day of differentiation (●) day11 (■) day 14 (▲) day 17 and (♦) day 20 and was obtained from two independent experiments (n=8). Horizontal lines and error bars represent mean ±SEM. Asterisk, significant difference between the $lysis_{50}$ values of ex vivo and in vitro cultured RNA+ CD71+ reticulocytes (p=0.02) using unpaired two-tailed student's *t*-test.

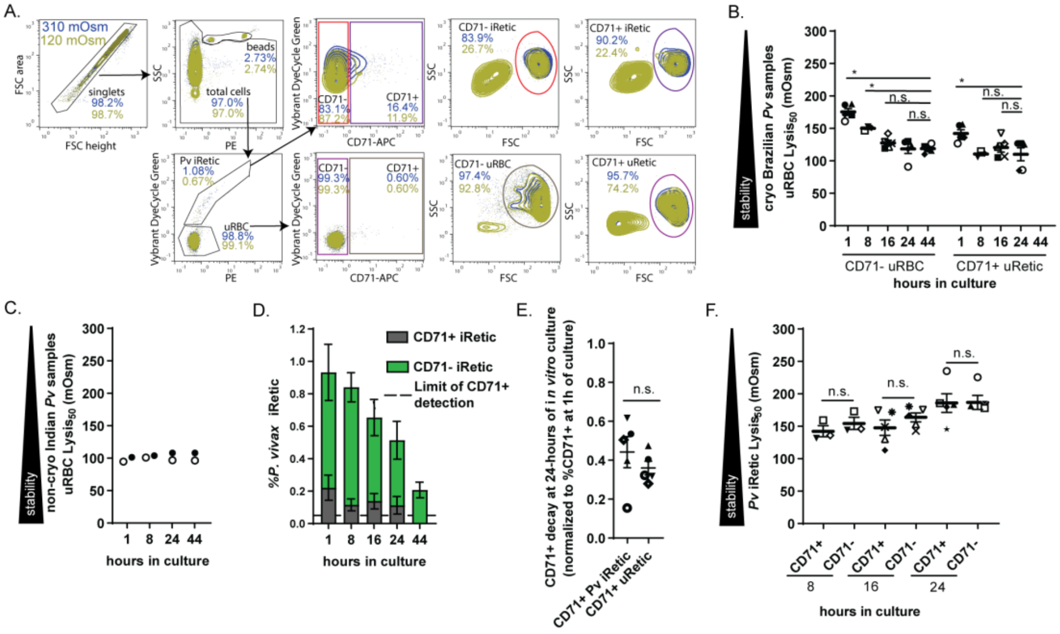

Supplementary Figure 3. Osmotic stability of *P. vivax* and *P. falciparum* infected RBCs. (a) Flow cytometry gating strategy for measuring hemolysis of CD71+ and CD71- *P. vivax*-infected reticulocytes and uninfected CD71+ reticulocytes and CD71- normocytes subpopulations present in cryopreserved Brazilian *P. vivax* samples. Gating strategy used to assess the osmotic stability of malaria infected RBC samples as reported in Figure 3, 4, and 5. (b) Uninfected CD71- and CD71+ uninfected RBC lysis$_{50}$ values for cryopreserved Brazilian *P. vivax* samples after 1- (n=5), 8- (n=3), 16- (n=5), 24- (n=5), and 44-hours (n=5) in culture. Unique symbols indicate biological replicates. Horizontal lines and error bars represent mean ±SEM. Asterisks and n.s., significant and no significant difference between CD71- uRBCs at 44-hours and 1-, 8-, 16-, and 24-hours of culture (1-hour p=0.001 8-hours p=0.003 16-hours p=0.5 24-hours p=0.9) and CD71+ uRetics at 24-hours and 1-, 8-, and16-hours of culture (1-hour p=0.02 8-hours p=0.9 16-hours p=0.6) respectively using unpaired two-tailed Dunnet's multiple comparisons test. (c) Uninfected RBCs from non-cryopreserved Indian *P. vivax* samples lysis$_{50}$ values (n=2) after 1-, 8-, 24-, and 44-hours of culture. (d) Mean parasitemia of cryopreserved Brazilian *P. vivax* samples after 1- (n=7), 8- (n=4), 16- (n=6) 24- (n=5)

and 44-hours (n=5) of culture. Green and gray bar segments represent the frequency of CD71- and CD71+ *P. vivax* infected reticulocytes respectively. SEM represented by error bars. Dashed line indicates the limit of detection for flow cytometry osmotic stability assays. (e) Percent loss of CD71+ infected and uninfected reticulocytes between 1 and 24-hours of culture (n=5). Unique symbols indicate biological replicates. Horizontal lines and error bars represent mean $\pm$SEM. n.s., no significant difference between decay of *P. vivax*-infected and uninfected CD71+ reticulocytes (p=0.4) using paired two-tailed student's *t*-test. (f) CD71+ and CD71- *P. vivax*-infected reticulocytes $lysis_{50}$ values for cryopreserved Brazilian *P. vivax* after 8- (n=4), 16- (n=6), and 24- (n=7) of culture. Unique symbols indicate biological replicates. Horizontal lines and error bars represent mean $\pm$SEM. n.s., no significant difference between CD71+ and CD71- *P. vivax*-infected reticulocyte $lysis_{50}$ (8-hours p=0.07, 16-hours p=0.3, 24-hours p=0.9) using paired two-tailed student's *t*-test.
