# Supplementary material for: Plasmodium vivax infection compromises reticulocyte stability
Source: Nat Commun. 2021 Mar 12;12:1629. doi: 10.1038/s41467-021-21886-x (PMC7955053; doi:10.1038/s41467-021-21886-x)
Supplement: Supplementary file 3 — Reporting Summary [file 41467_2021_21886_MOESM3_ESM.pdf]

nature research

Corresponding author(s): Manoj Duraisingh

Last updated by author(s): January 20, 2021

Reporting Summary

Nature Research wishes to improve the reproducibility of the work that we publish. This form provides structure for consistency and transparency in reporting. For further information on Nature Research policies, see our Editorial Policies and the Editorial Policy Checklist.

Statistics

For all statistical analyses, confirm that the following items are present in the figure legend, table legend, main text, or Methods section.

n/a	Confirmed	
☐	☒	The exact sample size (*n*) for each experimental group/condition, given as a discrete number and unit of measurement
☐	☒	A statement on whether measurements were taken from distinct samples or whether the same sample was measured repeatedly
☐	☒	The statistical test(s) used AND whether they are one- or two-sided<br>*Only common tests should be described solely by name; describe more complex techniques in the Methods section.*
☐	☒	A description of all covariates tested
☒	☐	A description of any assumptions or corrections, such as tests of normality and adjustment for multiple comparisons
☐	☒	A full description of the statistical parameters including central tendency (e.g. means) or other basic estimates (e.g. regression coefficient) AND variation (e.g. standard deviation) or associated estimates of uncertainty (e.g. confidence intervals)
☐	☒	For null hypothesis testing, the test statistic (e.g. *F*, *t*, *r*) with confidence intervals, effect sizes, degrees of freedom and *P* value noted<br>*Give P values as exact values whenever suitable.*
☒	☐	For Bayesian analysis, information on the choice of priors and Markov chain Monte Carlo settings
☒	☐	For hierarchical and complex designs, identification of the appropriate level for tests and full reporting of outcomes
☐	☒	Estimates of effect sizes (e.g. Cohen's *d*, Pearson's *r*), indicating how they were calculated

Our web collection on statistics for biologists contains articles on many of the points above.

Software and code

Policy information about availability of computer code

Data collection	MACSQuantify version 2.11, Zeiss AxioObserver.Z1
Data analysis	FlowJo (Version 10.4), Fiji Image J 1.52p, GraphPad Prism 8, Stata (version 16.0

For manuscripts utilizing custom algorithms or software that are central to the research but not yet described in published literature, software must be made available to editors and reviewers. We strongly encourage code deposition in a community repository (e.g. GitHub). See the Nature Research guidelines for submitting code & software for further information.

Data

Policy information about availability of data

All manuscripts must include a data availability statement. This statement should provide the following information, where applicable:

- Accession codes, unique identifiers, or web links for publicly available datasets
- A list of figures that have associated raw data
- A description of any restrictions on data availability

All data generated or analyzed during this study are included in this published article (and its supplementary information files). A reporting summary for this article is available as a Supplementary Information file. Source data are provided with this paper.

Field-specific reporting

Life sciences study design

All studies must disclose on these points even when the disclosure is negative.

Sample size	For flow cytometry analysis of >100,000 total events and the inclusion of cell counting beads (AccuCheck Counting Beads Thermo Fisher Scientific) provided for all cell populations above a frequency of 0.05% yielded accurate lysis counts as assessed by comparing lysis curves generated by flow cytometry and hemoglobin absorbance.<br><br>For counts of parasite IDC, by thin blood smears we counted at least 200 total infected erythrocytes which is consistent with similar studies (Lim, C. et al. Reticulocyte Preference and Stage Development of Plasmodium vivax Isolates. J Infect Dis. 214, 1081–1084 (2016) and Rangel, G. W. et al. Enhanced Ex Vivo Plasmodium vivax Intraerythrocytic Enrichment and Maturation for Rapid and Sensitive Parasite Growth Assays. Antimicrob. Agents Chemother. 62, (2018).)<br><br>For staging of in vitro differentiated RBCs, by cytospin we counted at least 200 total cells which is consistent with similar studies Daniels, D. E. et al. Comparing the two leading erythroid lines BEL-A and HUDEP-2. Haematologica 105, e389–e394 (2020). and Trakarnsanga, K. et al. An immortalized adult human erythroid line facilitates sustainable and scalable generation of functional red cells. Nat Commun 8, 14750 (2017).)
Data exclusions	No data was excluded.
Replication	Reproducibility was confirmed by performing independent experiments with a minimum of 2 biological replicates.
Randomization	Independent variables in this study are (i) osmolarity (ii) time (iii) sorbitol, alanine +/- furosemide treatment .<br>(i) osmolarity of lytic solutions was validated by vapor pressure osmometer (Wescor Vapro 5520)<br>(ii) we followed time using standard clocks<br>(iii) sorbitol, alanine, and furosemide dose was controlled with volumetric containers (e.g. serological pipettes) or mechanical pipettes calibrated on a regular basis.
Blinding	Experiments were not blinded as analyses derived from instrument-based measurements are less prone to user bias.

Reporting for specific materials, systems and methods

We require information from authors about some types of materials, experimental systems and methods used in many studies. Here, indicate whether each material, system or method listed is relevant to your study. If you are not sure if a list item applies to your research, read the appropriate section before selecting a response.

Materials & experimental systems

n/a	Involved in the study	
☐	☒	Antibodies
☐	☒	Eukaryotic cell lines
☒	☐	Palaeontology and archaeology
☒	☐	Animals and other organisms
☐	☒	Human research participants
☒	☐	Clinical data
☒	☐	Dual use research of concern

Methods

n/a	Involved in the study	
☒	☐	ChIP-seq
☐	☒	Flow cytometry
☒	☐	MRI-based neuroimaging

Antibodies

Antibodies used	α-CD71-APC clone AC102 Miltenyi Biotech 130-124-004, α-GypA-FITC clone 2b7 Stem Cell Technologies 60152FI, FITC-Phalloidin Thermo Fisher Scientific F432
Validation	- α-CD71-APC clone AC102 is noted by the manufacturer (Miltenyi Biotech) to detect human CD71 (transferrin receptor) (Loken, M. R. et al. (1987) Flow cytometric analysis of human bone marrow: I. Normal erythroid development. Blood 69: 255-263)<br>- α-GypA-FITC clone 2b7 is noted by the manufacturer to (Stem Cell Technologies) to detect human CD235 (GPA) (Paes BCMF et al. (2020) Generation of hematopoietic stem/progenitor cells with sickle cell mutation from induced pluripotent stem cell in serum-free system. In Press. DOI: 10.1016. (FC))<br>- FITC-Phalloidin is noted by the manufacturer (Thermo Fisher) to detect human F actin ((-)-Doliculide, a new macrocyclic depsipeptide enhancer of actin assembly. Bai R, Covell DG, Liu C, Ghosh AK, Hamel E J Biol Chem (2002) 277:32165-32171)

Eukaryotic cell lines

Policy information about cell lines

Cell line source(s)	Pf3D7 clone P2G12 was obtained from the laboratory of Matthias Marti. The Marti lab is where Pf3D7 clone P2G12 originated.
Authentication	no formal authentication was performed. cell line confirmed to characteristic growth and morphology for the parasite.
Mycoplasma contamination	mycoplasma contamination testing was not performed.
Commonly misidentified lines (See ICLAC register)	No commonly misidentified cell lines were used in the study.

Human research participants

Policy information about studies involving human research participants

Population characteristics	Anonymized discarded human bone marrow samples were negative for blasts or dis-erythropoietic conditions.<br><br>Brazilian and Indian clinical P. vivax samples were collected from individuals infected with P. vivax infection confirmed by microscopy.<br><br>We do not have covariate-relevant population chararateristicis for the Brazilian and India human research participants. We obtain de-identified parasite samples from these individuals. The human characteristics are not relevant to the study.
Recruitment	Anonymized discarded human bone marrow samples negative for blasts or dis-erythropoietic conditions were obtained from Boston Children's Hospital.<br><br>Brazilian clinical P. vivax samples were collected in the town of Mâncio Lima, Acre State, and processed as described elsewhere 57. The collections were performed in the context of a randomized, open-label clinical trial (NCT02691910). Indian P. vivax samples were collected with similar protocols but using CF11 for leukodepletion, as described previously 58. This occurred at Goa Medial College and Hospital in Bambolim, Goa, in conjunction with the Malaria Evolution in South Asia International Center of Excellence in Malaria Research and the University of Washington.
Ethics oversight	For hereditary xerocytosis, bone marrow aspirate, and Brazilian and Indian clinical P. vivax samples, we confirm that all relevant ethical regulations were complied with. For hereditary xerocytosis and human bone marrow samples negative for blasts or dis-erythropoietic conditions, informed consent was obtained for all patients and samples were collected under the approval of the Boston Children's Hospital Institutional Review Board (IRB04-02-017R) and were de-identified for use in this study. We obtained human IRB waivers for the use of the de-identified parasite samples from the Harvard School of Public Health Office of Human Research Administration for Brazil (IRB21410-101) and for India (IRB17-1071). For Brazilian clinical P. vivax samples, informed consent was obtained from all patients. Study protocols for Brazilian parasite sample collection were approved by the IRB of the Institute of Biomedical Sciences, University of São Paulo, Brazil (IRB 1169/CEPSH, 2014). For Indian P. vivax samples, informed consent was obtained from all patients. Study protocols for Indian parasite sample collection were approved by the ethics boards at Goa Medical College and Hospital (GMC) (no number assigned), University of Washington (42271), the Division of Microbiology and Infectious Diseases of the US National Institutes of Health (NIH) (11-0074) and the Government of India Health Ministry Screening Committee (HMSC) for collection of the Indian isolates.

Note that full information on the approval of the study protocol must also be provided in the manuscript.

Flow Cytometry

Plots

Confirm that:

- [x] The axis labels state the marker and fluorochrome used (e.g. CD4-FITC).
- [x] The axis scales are clearly visible. Include numbers along axes only for bottom left plot of group (a 'group' is an analysis of identical markers).
- [x] All plots are contour plots with outliers or pseudocolor plots.
- [x] A numerical value for number of cells or percentage (with statistics) is provided.

Methodology

Sample preparation	Peripheral blood, RBCs were washed and lysed in osmotic stability assays before flow cytometry analysis. Bone marrow aspirates, RBC progenitors were enriched using percoll gradients washed stained for flow cytometry and lysed in osmotic stability assay before being analyzed by flow cytometry. For all RBCs coming from culture, RBCs were washed, fluorescently stained when applicable, lysed in osmotic stability assay before being analyzed by flow cytometry.
Instrument	Miltenyi MACSQuant Analyzer 10 equipped with 405-nm, 488-nm, and 638-nm lasers.

Software	Flow cytometry data was collected using MACSQuantify (version 2.11) and data analyzed with FlowJo (Version 10.4).
Cell population abundance	no sorting was done for this study.
Gating strategy	Single RBC measurements were selected by FSC-H/ FSC-A gating. No-lysis control conditions were used to set RBC FSC/ SSC gates and were drawn so that 90% of cells were included for further analysis. Gating on RBC precursors gates were set based on positive signal for DNA dye Vybrant DyeCycle Violet. CD71+ reticulocytes gates were set based on positive signal for CD71-APC ab and RNA dye Thiazole Orange. CD71- RNA+ reticulocyte gates were set based on negative signal for CD71-APC ab and positive signal for RNA dye Thiazole Orange. CD71- RNA- normocytes gates were based on negative signal for CD71-APC antibody and RNA dye Thiazole Orange. CD71+ malaria infected RBCs gates were based on positive signal for DNA dye Vybrant DyeCycle Green and CD71-APC ab. CD71- malaria infected RBC gates were based on postive signal for DNA dye Vybrant DyeCylce Green and negative signal for CD71-APC ab. Gating strategy for identification of (i) lysed cells (ii) reticulocyte and red cell precursor and (iii) plasmodium infected cells are shown in supplemental figures 1, 2A, and 3A.

☒ Tick this box to confirm that a figure exemplifying the gating strategy is provided in the Supplementary Information.
